# Comparison between sufentanil and hydromorphone for postoperative analgesia after cesarean section: a propensity-score matched analysis

**DOI:** 10.3389/fmed.2026.1834000

**Published:** 2026-07-10

**Authors:** Hui Wu, Weigang Zhou, Yu Du, Jiao Chen

**Affiliations:** 1 Department of Anesthesiology, Hanzhong Central Hospital, Hanzhong, Shaanxi, China; 2 Department of Anesthesiology, Beijing Anzhen Nanchong Hospital of Capital Medical University, The Affiliated Nanchong Central Hospital of North Sichuan Medical College, Nanchong, Sichuan, China; 3 Department of Laboratory Medicine, Hanzhong Central Hospital, Hanzhong, Shaanxi, China

**Keywords:** cesarean section, hydromorphone, patient controlled analgesia (PCA), propensity score matching (PSM), sufentanil

## Abstract

**Background:**

Cesarean delivery often causes significant postoperative pain, which can affect recovery and increase the risk of chronic pain and prolonged opioid use. This study compared commonly used hydromorphone and sufentanil regarding postoperative outcomes after cesarean delivery.

**Method:**

This multi-center retrospective cohort study, conducted across multiple centers in China between June 2022 and June 2025, included patients undergoing elective cesarean sections who received patient-controlled analgesia (PCA) with either hydromorphone or sufentanil. Postoperative pain, measured by the numerical rating scale (NRS), was the primary outcome. Secondary outcomes included use of rescue opioid use, incidence of postoperative nausea and vomiting (PONV), and hospitalization costs. Propensity score matching (PSM) was used to balance baseline characteristics, and regression models were applied to compare primary and secondary outcomes between groups.

**Result:**

Among 10,608 eligible patients, PSM yielded 2,682 matched pairs with good covariate balance. After matching, median NRS scores on day 1 were 4 [3–5] in sufentanil group, 4 [4–5] in hydromorphone group; on day 3, the score was 3 [2–3] in both groups. Matched pair-aware analyses showed no significant between-group differences in pain (day 1 mean difference 0.002, 95% CI −0.056 to 0.060, *P* = 0.945; day 3 mean difference 0.001, 95% CI −0.047 to 0.049, *P* = 0.960). Observed PONV was lower with sufentanil (4.88% vs. 7.38%, *P* < 0.001), but this was not statistically significant in matched pair-aware analysis (OR 0.815, 95% CI 0.235–2.830, *P* = 0.747). Rescue opioid use also did not differ significantly (OR 0.564, 95% CI 0.081–3.932, *P* = 0.563). Costs (in Renminbi) were lower with sufentanil in matched analyses: total cost mean difference −125.481 (95% CI −193.189 to −57.773), anesthesia cost −82.107 (95% CI −86.245 to −77.969), and PCA cost −82.218 (95% CI −85.298 to −79.138), all *P* < 0.001.

**Conclusion:**

Hydromorphone and sufentanil provided comparable postoperative analgesia after elective cesarean section. Sufentanil was associated with lower analgesia-related and total costs, while differences in PONV and rescue opioid use were not statistically significant in matched pair-aware analyses.

## Introduction

Patients undergoing cesarean delivery may experience severe postoperative pain (1). Acute pain after surgery not only affects immediate recovery but also contributes to the risk of chronic pain, prolonged opioid exposure, and other adverse outcomes (2, 3). Effective and sufficient analgesia in the first 24 postoperative hours after cesarean section is therefore essential for maternal recovery (4).

Preemptive analgesia can potentially reduce postoperative pain, opioid consumption, and postoperative nausea and vomiting while delaying rescue analgesia (5). Hydromorphone and sufentanil are common opioids for post-cesarean patient-controlled analgesia (PCA). Hydromorphone provides strong and sustained analgesia and is associated with relatively fewer adverse reactions due to its hydrophilicity, with literature supporting its efficacy even when administered via alternative routes such as the intrathecal route (6, 7). Sufentanil is a potent opioid analgesic with high selectivity for mu-opioid receptors, offering rapid onset, cardiovascular stability, and a wide therapeutic margin at a relatively low cost (8, 9).

Although enhanced recovery after surgery (ERAS) protocols emphasize multimodal opioid-sparing analgesia (10), intravenous patient-controlled analgesia (IV-PCA) remains widely used in many Chinese obstetric centers (11, 12). This is partly due to practical barriers to neuraxial morphine administration (e.g., limited availability of preservative-free morphine for intrathecal use in some regions), inconsistent access to ultrasound-guided truncal blocks, and institutional familiarity with IV-PCA workflows (10, 13). Optimizing IV-PCA opioid selection remains a clinically relevant question.

Prior studies suggested comparable analgesic efficacy between these opioids when given in equivalent PCA doses (14, 15). However, side-effect profiles differ as morphine-class opioids are often thought to cause more nausea/vomiting than fentanyl analogues, while fentanyl-class drugs may require more frequent dosing or higher total doses due to shorter duration of action (11). A recent trial found that adding hydromorphone to sufentanil PCA improved analgesia and mood without increasing adverse effects, but it did not compare hydromorphone alone with sufentanil alone (4).

To our knowledge, few studies have directly contrasted postoperative sufentanil vs. hydromorphone in cesarean patients. This study evaluates postoperative outcomes in matched cohorts of cesarean patients receiving PCA by either hydromorphone or sufentanil.

## Materials and method

### Study design

This multi-center retrospective cohort study was conducted according to the Strengthening the Reporting of Observational Studies in Epidemiology (STROBE) statement (16). Ethical approval was obtained from the Ethics Committee of Hanzhong Central Hospital (23 [2026]). Informed consent was waived as this study was conducted retrospectively using anonymized patient data.

### Participants

Patients who underwent elective cesarean sections with postoperative patient-controlled analgesia (PCA) were included in the study. The exclusion criteria were age below 20 years, gestational age below 32 weeks, and had more than five missing variables.

Patients were grouped by PCA opioid (hydromorphone vs. sufentanil). Baseline demographic, perioperative, and analgesia-related variables were extracted. PCA solutions were formulated at a concentration of 1 μg/ml for sufentanil and 40 μg/ml for hydromorphone. The default pump settings consisted of a background infusion of 1–1.5 ml/h for sufentanil and 1.5–2.0 ml/h for hydromorphone, with a fixed 3 ml bolus for both groups. This delivered a background dose of 1–1.5 μg/h for sufentanil and 60–80 μg/h for hydromorphone, with bolus doses of 3 μg and 120 μg, respectively, and a 10-min lockout interval. These doses were selected based on established empirical clinical use (11).

### Outcomes

The primary outcome was postoperative pain intensity assessed by the numerical rating scale (NRS) at rest each morning on postoperative day 1 and at the earlier of postoperative day 3 or hospital discharge. PONV was recorded as incidence within 48 h. Secondary outcomes included the need for opioid rescue analgesia and costs. Cost endpoints included total hospitalization cost, total anesthesia cost (including perioperative and postoperative analgesia), and PCA-specific cost. Cost data were computed from hospital billing records in local currency (Renminbi, RMB).

### Missing data imputation

Variables with missing values were imputed using predictive mean matching via the package “mice”(17). Binary and categorical variables were converted to factors before imputation. One completed dataset was used for downstream analysis. Pooled estimates across multiple imputed datasets were not generated.

### Propensity score matching (PSM)

Propensity score matching was performed to adjust confounders between groups using R package “MatchIt” (Version 4.5.5) (18). Propensity scores for receiving sufentanil versus hydromorphone were estimated using a logistic regression model including age, BMI, type of anesthesia, anesthesia duration, operation duration, parity, gestational weeks, hypertension, diabetes, uterine scar, and anxiety/depression. Patient height and weight were not included in the propensity score model due to high correlation with BMI. Patients were matched 1:1 using nearest neighbor matching with a caliper of 0.02 and standardized caliper, without replacement (19). Covariate balance before and after matching was assessed using standardized mean differences (SMD), with an absolute SMD < 0.1 considered acceptable.

### Statistical analysis

Analyses were performed using R (version 4.5.2). Normally distributed continuous data were presented as mean ± standard deviation (SD), and non-normal data were presented as median [interquartile range]. Categorical data were presented as frequency (percentage). The Shapiro-Wilk test was used to assess the normality of continuous variables. Normal and non-normal variables were analyzed using the *t*-test and the Mann-Whitney U test, respectively. The Chi-square test was used to analyze categorical variables. Regression models adjusted for baseline covariates, including the origin institution to account for potential center-level effects, were fitted to estimate the effect of PCA drug on each outcome. Linear regression was used for continuous outcomes, and logistic regression was used for binary outcomes before matching. In the matched cohort, pair-aware analyses were performed using cluster-robust standard errors for continuous outcomes and conditional logistic regression stratified by matched pair for binary outcomes.

## Result

**FIGURE 1:**
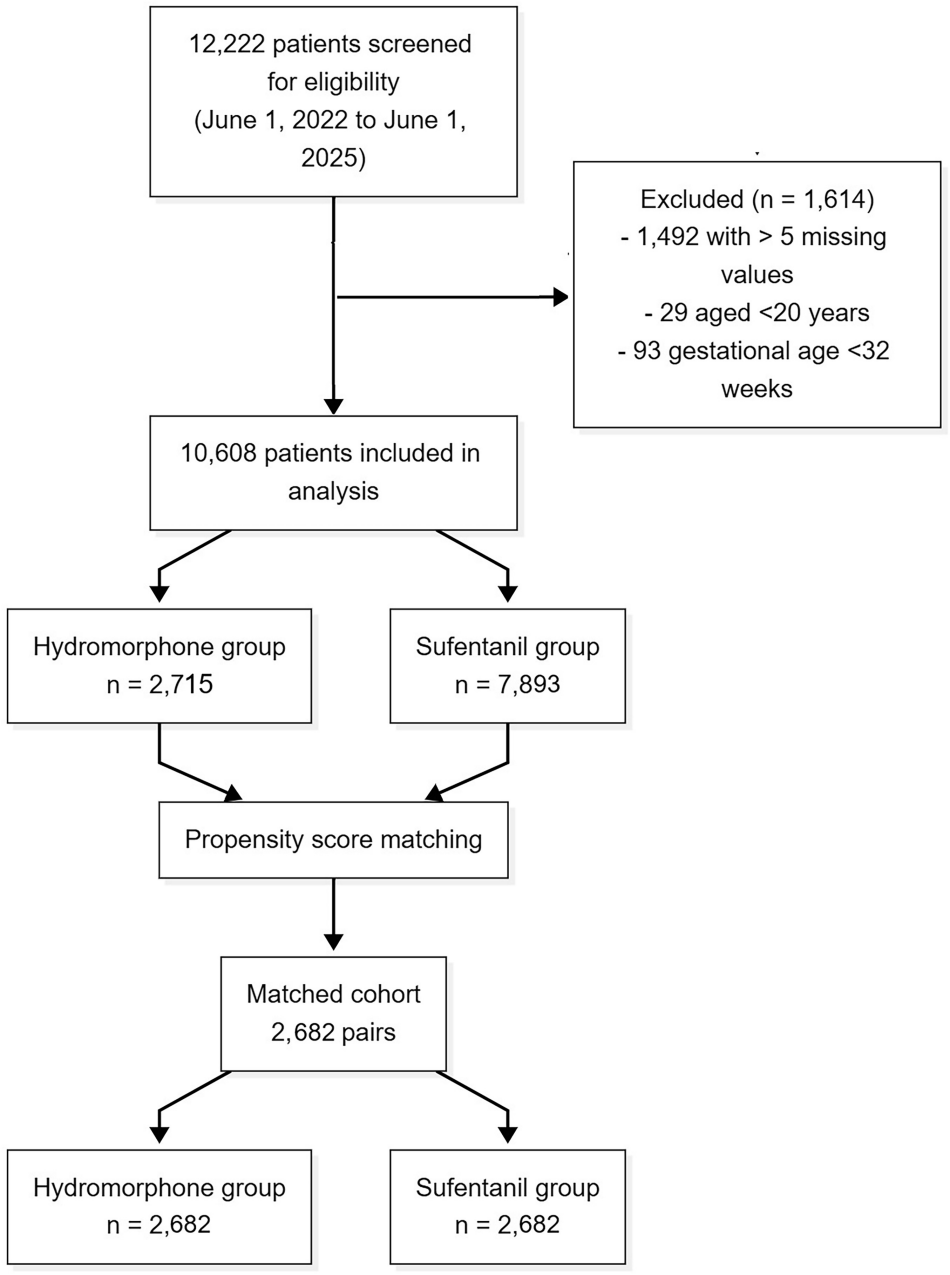
Patient flow chart.

A total of 12,222 patients who underwent cesarean section between June 1, 2022, and June 1, 2025, were screened for eligibility. After excluding 1,492 records with six or more missing values, 29 patients aged below 20 years, and 93 patients with gestational age less than 32 weeks, 10,608 patients were included in the analysis. Before propensity score matching (PSM), baseline characteristics were generally comparable between the hydromorphone and sufentanil groups, although gestational weeks differed significantly between groups. After PSM, 2,682 patients remained in each group, and all baseline covariates were well balanced, with all available SMDs below 0.1 and all *P*-values above 0.1 (Table 1). A patient flow chart is presented in Figure 1. Correlations among study variables are shown in Supplementary Figure 1.

**TABLE 1:** Patient baseline information.

Characteristics		Before PSM				After PSM			
Drug		Hydromorphone (*N* = 2715)	Sufentanil (*N* = 7893)	*P*	SMD	Hydromorphone (*N* = 2682)	Sufentanil (*N* = 2682)	*P*	SMD
Age (years)		30 [28–34]	30 [28–34]	0.446	−0.037	30 [28–34]	31 [28–34]	0.695	0.019
Weight (kg)		72 [71–73]	72 [71–73]	0.544	N.A.^a^	72 [71–73]	72 [71–73]	0.99	N.A.^a^
Height (cm)		163 [161–165]	163 [161–165]	0.934	N.A.^a^	163 [161–165]	163 [161–165]	0.816	N.A.^a^
BMI		27.1 [26.3–27.9]	27.1 [26.3–27.9]	0.772	0.001	27.1 [26.3–27.9]	27.1 [26.3–27.9]	0.773	−0.008
Type of anesthesia	General	25 (0.92%)	92 (1.17%)	0.292	0.001	25 (0.93%)	33 (1.23%)	0.291	−0.028
	Neuraxial	2690 (99.08%)	7801 (98.83%)		−0.001	2657 (99.07%)	2649 (98.77%)		0.028
Anesthesia duration		65 [55–75]	65 [55–75]	0.461	−0.003	65 [55–75]	65 [55–75]	0.923	0.002
Operation duration		45 [40–58]	45 [40–56]	0.202	−0.061	45 [40–57]	45 [40–57]	0.85	0.011
Parity	Multiparous	1306 (48.10%)	3874 (49.08%)	0.379	0.017	1286 (47.95%)	1276 (47.58%)	0.785	−0.007
	Primiparous	1409 (51.90%)	4019 (50.92%)		−0.017	1396 (52.05%)	1406 (52.42%)		0.007
Gestational weeks		38 [38–39]	38 [38–39]	0.006	−0.055	38 [38–39]	38 [38–39]	0.428	−0.028
Hypertension		38 [38–39]	38 [37–39]	0.705	−0.009	419 (15.62%)	419 (15.62%)	1	0
Diabetes		421 (15.51%)	1200 (15.20%)	0.5	−0.003	477 (17.79%)	467 (17.41%)	0.72	−0.01
Uterus scar		485 (17.86%)	1365 (17.29%)	0.064	0.037	643 (23.97%)	634 (23.64%)	0.773	−0.008
Anxiety and depression		647 (23.83%)	2022 (25.62%)	0.385	0.017	1 (0.04%)	0 (0.0%)	0.317	−0.009
Institution	Hanzhong	1557 (57.35%)	4403 (55.78%)	0.156	−0.019	1536 (57.27%)	1497 (55.82%)	0.283	−0.029
	Nanchong	1158 (42.65%)	3490 (44.22%)		0.019	1146 (42.73%)	1185 (44.18%)		0.029

Data are presented as median [interquartile ranges] or n (%).

*^a^*Weight and height were not included in the propensity score matching model; therefore, standardized mean differences were not calculated for these variables. PSM, propensity score matching; SMD, standardized mean difference; BMI, body mass index.

Postoperative pain intensity, assessed using the numerical rating scale (NRS), was similar between groups in the observed data. After matching, the median NRS score on postoperative day 1 was 4 [IQR 4–5] in both groups, and the median score on postoperative day 3 was 3 [IQR 2–3] in both groups (Table 2). Consistent with these descriptive findings, the regression analyses showed no significant difference in pain scores between sufentanil and hydromorphone. In the pre-matching multivariable analysis, the mean differences were 0.011 (95% CI −0.037 to 0.058, *P* = 0.663) for postoperative day 1 and 0.013 (95% CI −0.026 to 0.053, *P* = 0.517) for postoperative day 3. In the matched pair-aware analysis, the corresponding mean differences were 0.002 (95% CI −0.056 to 0.060, *P* = 0.945) and 0.001 (95% CI −0.047 to 0.049, *P* = 0.960), indicating no clinically or statistically meaningful difference in postoperative pain control between sufentanil- and hydromorphone-based PCA regimens (Table 3).

**TABLE 2:** Observed outcomes in the sufentanil and hydromorphone groups.

Outcomes	Before PSM			After PSM		
	Hydromorphone (*N* = 2715)	Sufentanil (*N* = 7893)	*P*	Hydromorphone (*N* = 2682)	Sufentanil (*N* = 2682)	*P*
NRS-D1	4 [4–5]	4 [3–5]	0.792	4 [4–5]	4 [3–5]	0.934
NRS-D3	3 [2–3]	3 [2–3]	0.813	3 [2–3]	3 [2–3]	0.778
PONV	200 (7.37%)	413 (5.23%)	<0.001	198 (7.38%)	131 (4.88%)	<0.001
Total cost (RMB)	9496.2 [8715.8–10364.2]	9410.2 [8576.2–10246.1]	<0.001	9499.1 [8712.3–10366.9]	9404.1 [8556.1–10234.2]	<0.001
Anesthesia cost (RMB)	1080 [1000–1100]	980 [920–1020]	<0.001	1080 [1000–1100]	980 [920–1020]	<0.001
PCA cost (RMB)	370 [290–450]	290 [230–350]	<0.001	370 [290–450]	290 [230–350]	<0.001
Rescue opioids	117 (4.31%)	405 (5.13%)	0.088	116 (4.33%)	146 (5.44%)	0.057

Values are observed (unadjusted) outcomes. *P*-values are from groupwise comparisons (Mann-Whitney U for continuous variables, chi-square for categorical variables).

**TABLE 3:** Effect sizes of sufentanil versus hydromorphone.

Outcomes		Before PSM		After PSM	
	Effect measure	Effect size (95% CI)	*P*	Effect size (95% CI)	*P*
NRS-D1	Mean difference	0.011 (−0.037, 0.058)	0.663	0.002 (−0.056, 0.060)	0.945
NRS-D3	Mean difference	0.013 (−0.026, 0.053)	0.517	0.001 (−0.047, 0.049)	0.960
PONV	Odds ratio	0.689 (0.579, 0.822)	<0.001	0.815 (0.235, 2.830)	0.747
Total cost (RMB)	Mean difference	−120.056 (−174.513, −65.598)	<0.001	−125.481 (−193.189, −57.773)	<0.001
Anesthesia cost (RMB)	Mean difference	−81.893 (−85.149, −78.638)	<0.001	−82.107 (−86.245, −77.969)	<0.001
PCA cost (RMB)	Mean difference	−81.488 (−83.823, −79.153)	<0.001	−82.218 (−85.298, −79.138)	<0.001
Rescue opioids	Odds ratio	1.197 (0.973, 1.484)	0.094	0.564 (0.081, 3.932)	0.563

Effect sizes are reported for sufentanil relative to hydromorphone. Mean differences less than 0 indicate lower values in the sufentanil group, and odds ratios less than 1 indicate lower odds in the sufentanil group. Before PSM, effect estimates were obtained from multivariable regression in the full cohort; after PSM, effect estimates were obtained from matched pair-aware models in the propensity score-matched cohort. *P*-values less than 0.05 were considered statistically significant. PSM, propensity score matching; CI, confidence interval; NRS, numeric rating scale; PONV, postoperative nausea and vomiting; PCA, patient-controlled analgesia.

For secondary outcomes, the observed incidence of postoperative nausea and vomiting (PONV) was lower in the sufentanil group both before matching (5.23% vs. 7.37%) and after matching (4.88% vs. 7.38%) (Table 2). In the pre-matching multivariable analysis, sufentanil was associated with lower odds of PONV (OR 0.689, 95% CI 0.579–0.822, *P* < 0.001). However, in the matched pair-aware analysis, the direction of effect remained favorable but statistical significance was not retained (OR 0.815, 95% CI 0.235–2.830, *P* = 0.747) (Table 3). The proportion of patients requiring rescue opioids was low in both groups and did not differ meaningfully in either the descriptive comparisons or the regression analyses. The pre-matching multivariable model yielded an OR of 1.197 (95% CI 0.973–1.484, *P* = 0.094), and the matched pair-aware model yielded an OR of 0.564 (95% CI 0.081–3.932, *P* = 0.563) (Table 3).

Analgesia-related costs differed consistently between groups. In the observed matched cohort, the sufentanil group had lower PCA-specific cost, lower anesthesia-related cost, and lower total hospitalization cost than the hydromorphone group (Table 2). These differences were confirmed in the regression analyses. In the pre-matching multivariable analysis, the mean differences for sufentanil versus hydromorphone were −120.056 (95% CI −174.513 to −65.598, *P* < 0.001) for total cost, −81.893 (95% CI −85.149 to −78.638, *P* < 0.001) for anesthesia cost, and −81.488 (95% CI −83.823 to −79.153, *P* < 0.001) for PCA cost. In the matched pair-aware analysis, the corresponding mean differences were −125.481 (95% CI −193.189 to −57.773, *P* < 0.001), −82.107 (95% CI −86.245 to −77.969, *P* < 0.001), and −82.218 (95% CI −85.298 to −79.138, *P* < 0.001), respectively (Table 3).

## Discussion

In this multi-center retrospective study, data from 10,608 patients who underwent cesarean section were analyzed. After propensity score matching to adjust for confounding factors, we found no difference in efficacy of pain management between sufentanil and hydromorphone. PONV was numerically lower with sufentanil before and after matching, but the matched pair-aware model did not retain statistical significance. The use of hydromorphone was associated with higher anesthesia-related cost during hospital stays.

Postoperative pain scores (NRS) on both the first and third days were comparable between patients receiving hydromorphone or sufentanil. These results are consistent with prior reports that patient-controlled hydromorphone and sufentanil can provide similar analgesia (15). Ouyang et al. reported that sufentanil combined with hydromorphone could improve postoperative pain compared with sufentanil alone; however, this differs from our study in study design and intervention (4).

The association between opioid choice and PONV was not fully robust across analytic methods. Although the pre-matching multivariable model suggested lower odds of PONV with sufentanil, this finding was not statistically significant in the matched pair-aware analysis, despite a similar protective direction of effect. Accordingly, any apparent antiemetic advantage of sufentanil should be interpreted as hypothesis-generating rather than conclusive. This finding differs from a randomized study reporting no significant difference in PONV when hydromorphone was added to sufentanil, which may reflect differences in intervention strategy and study design (4). Nevertheless, the observed direction is pharmacologically plausible, as fentanyl-class opioids such as sufentanil may be less emetogenic than morphine-like opioids in some clinical settings (12, 14).

The patient-controlled analgesia costs were lower in the sufentanil group, accompanied by reduced anesthesia-related costs, whereas the difference in total hospitalization costs was small in absolute magnitude. A recent PSM study similarly found that combining an additional agent greatly increased analgesic cost without improving efficacy (20). Overall, sufentanil was associated with lower analgesia-related costs while providing postoperative pain control comparable to hydromorphone, with no meaningful difference in total hospitalization costs.

This study has several strengths. The large multicenter cohort enhances the generalizability of the findings within similar clinical settings. In addition, propensity score matching improved baseline comparability between treatment groups and reduced measured confounding, thereby strengthening the validity of the between-group comparisons. Another important strength is the consistent finding that sufentanil was associated with lower analgesia-related costs while providing postoperative pain control comparable to hydromorphone. These results suggest that, in this setting, sufentanil may offer a clinically efficient and economically favorable PCA strategy.

There were limitations as well. Firstly, the retrospective design prevents causal inference, and several important confounders — intrathecal opioid dose, intraoperative opioid consumption, NSAID and acetaminophen use, TAP block utilization, surgical complexity, and pre-existing chronic pain — were not available and cannot be addressed by propensity score matching. Second, strict pharmacological equipotency between sufentanil and hydromorphone at the ratios employed is not definitively established; if doses are not truly equianalgesic, efficacy, adverse effect, and cost comparisons may be systematically biased. Third, only NRS pain scores at rest were available; dynamic pain is more functionally relevant after cesarean section. Fourth, ERAS-specific recovery outcomes were not captured. Fifth, neonatal outcomes, breastfeeding safety profiles, and breast milk transfer data were not assessed; sufentanil and hydromorphone may differ in mammary transfer kinetics. Sixth, formal sensitivity analyses were not performed, and missing data were addressed using a single imputed dataset rather than pooled multiple imputation. Seventh, median NRS scores of 3–4 may create a floor effect limiting detection of small between-group differences. Furthermore, the relatively high mismatch rate during PSM is an artifact of the 1:1 matching design and the size imbalance between cohorts; unmatched cases consisted primarily of surplus sufentanil patients, preserving the representativeness of the smaller group.

## Conclusion

Among women undergoing cesarean section with postoperative IV-PCA, sufentanil and hydromorphone provided comparable postoperative pain control. Sufentanil was consistently associated with statistically lower analgesia-related, anesthesia-related, and total hospitalization costs, although the absolute differences may be of limited clinical or economic significance. For PONV, the estimated direction favored sufentanil, but statistical significance was not retained in matched pair-aware analysis; therefore, this association should be interpreted with caution and confirmed in prospective studies.

## Data Availability

The raw data supporting the conclusions of this article will be made available by the authors, without undue reservation.

## Supplementary material

Supplementary Figure 1 Correlation matrix of study variables. Pearson correlation coefficients are shown for baseline characteristics, postoperative outcomes, and cost variables. Color intensity and direction indicate the strength and direction of correlation (red = positive, blue = negative).
