# Supplementary figures and images for: Comparison between sufentanil and hydromorphone for postoperative analgesia after cesarean section: a propensity-score matched analysis
Source: Front Med (Lausanne). 2026 Jul 10;13:1834000. doi: 10.3389/fmed.2026.1834000 (PMC13396168; doi:10.3389/fmed.2026.1834000)

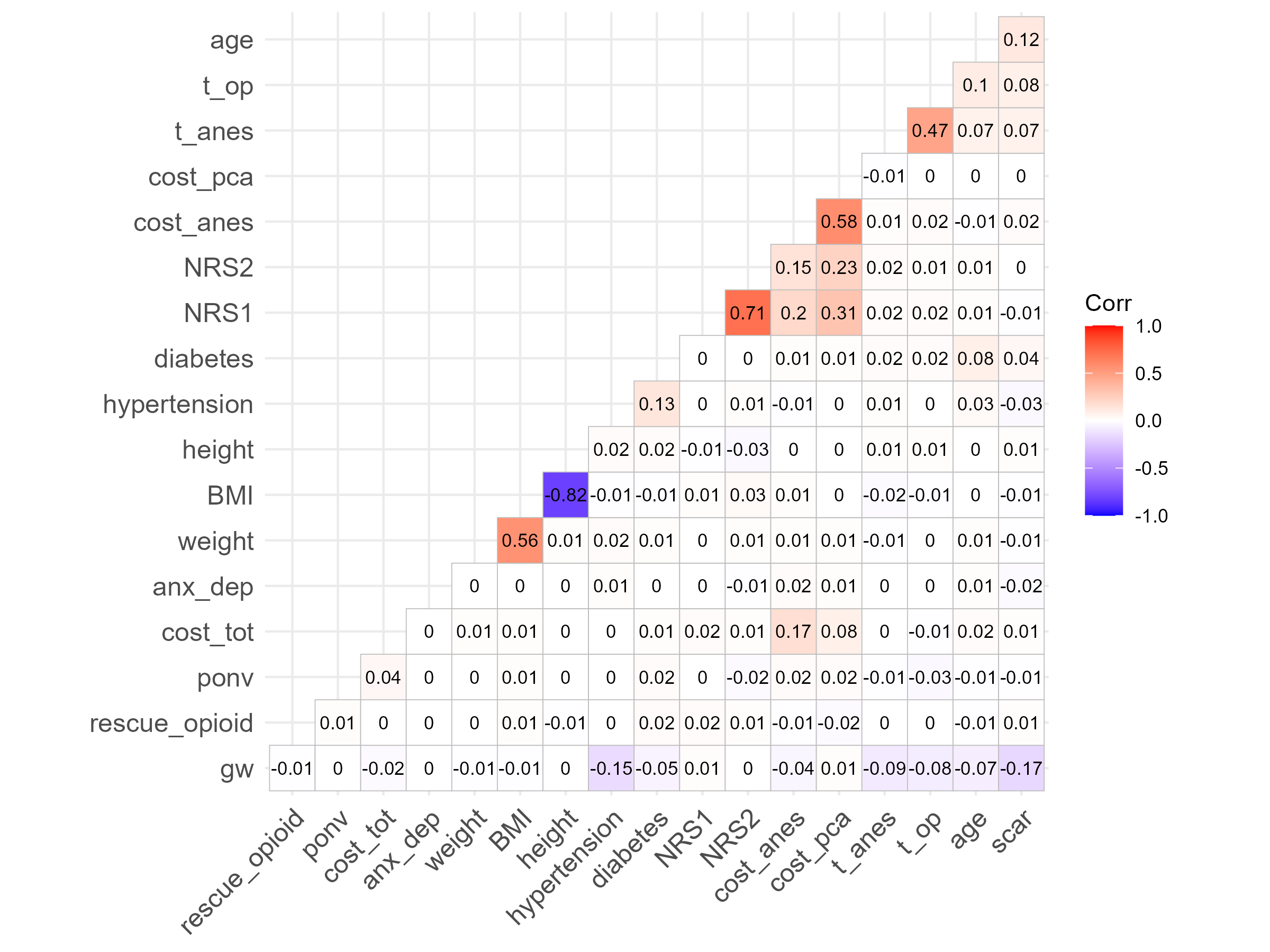

Supplement: Supplementary Figure 1 — Correlation matrix of study variables. Pearson correlation coefficients are shown for baseline characteristics, postoperative outcomes, and cost variables. Color intensity and direction indicate the strength and direction of correlation (red = positive, blue = negative). [file Image_1.png]
